# Ruxolitinib cream for the treatment of granuloma annulare

**DOI:** 10.1016/j.jdcr.2024.05.030

**Published:** 2024-06-05

**Authors:** Austin J. Piontkowski, Nancy Wei, Aisha Mumtaz, Nicholas Gulati

**Affiliations:** Department of Dermatology, Icahn School of Medicine at Mount Sinai, New York, New York

**Keywords:** granuloma annulare, JAK inhibitor, Janus kinase inhibitor, ruxolitinib

## Introduction

Granuloma annulare is a rare granulomatous inflammatory skin condition that can either be generalized or localized. Generalized granuloma annulare (GGA) is characterized by widespread annular plaques, while localized forms tend to present as annular or circinate erythematous to flesh-colored papules and plaques.^1^ Conventional treatment includes topical or intralesional corticosteroids, though their efficacy can be limited, especially for disseminated disease. As such, systemic therapy with hydroxychloroquine is often considered first-line therapy for GGA.^2^ Nonetheless, the rarity of the disease and limited research into treatment strategies pose unique challenges to management, particularly for refractory cases. Herein, we present the case of a 51-year-old female with refractory, chronic GGA who achieved complete resolution with topical ruxolitinib cream.

## Case history

**Fig 1:**
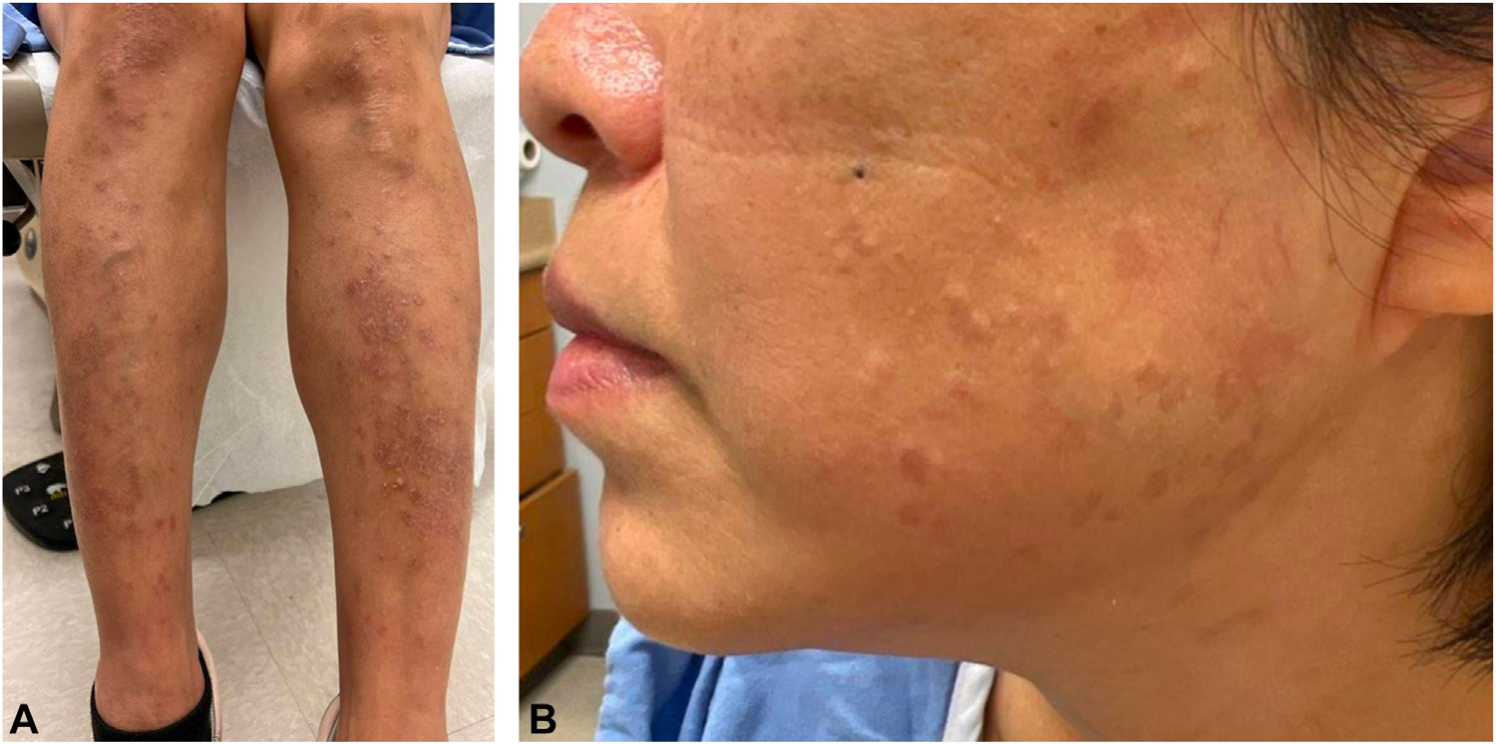
Erythematous, annular plaques, and papules on the bilateral lower extremities (**A**) and face (**B**) prior to treatment with ruxolitinib 1.5% cream.

**Fig 2:**
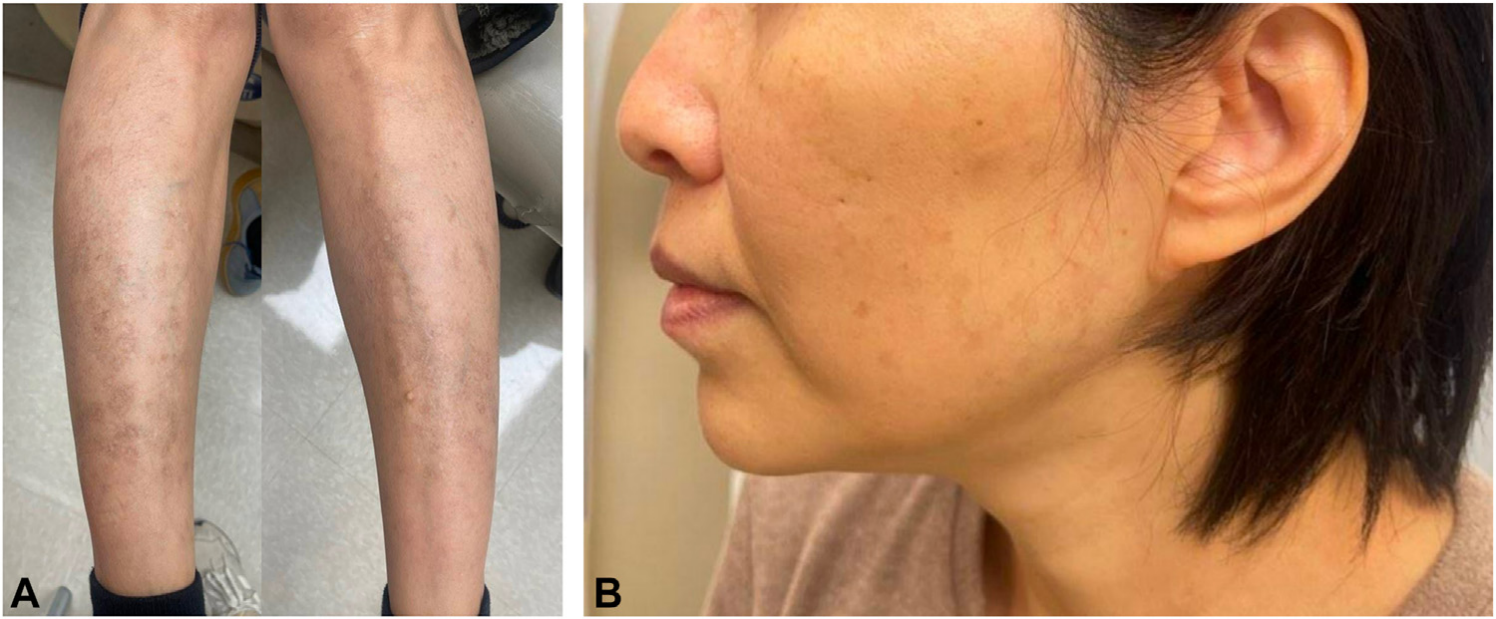
Complete response to 12 weeks of treatment with ruxolitinib 1.5% cream, with residual hyperpigmented patches consistent with resolution of prior plaques and papules on the bilateral lower extremities (**A**) and face (**B**).

A 51-year-old female with no significant past medical history presented for reassessment of asymptomatic erythematous, annular plaques, and papules predominantly on the face and lower extremities. The lesions initially manifested 2 years prior, and a skin biopsy at that time revealed granulomatous inflammation with the presence of mucin on histology. While cutaneous sarcoidosis was considered, the clinical morphology and histological presence of mucin favored a diagnosis of granuloma annulare. Pulmonary workup, including pulmonary function tests and chest imaging, revealed no evidence of sarcoidosis. The patient failed initial topical treatment with twice-daily application of betamethasone dipropionate 0.05% cream for several months for suspected granuloma annulare. The patient was then prescribed tacrolimus 0.1% ointment, but no improvement was observed after 6 weeks of twice-daily use. Intralesional triamcinolone injections were also attempted, but proved to be minimally effective. Subsequently, the patient was started on hydroxychloroquine 200 mg twice daily without improvement over the course of a year, prompting a repeat biopsy that demonstrated nonspecific granulomatous and lymphocytic inflammation. Pulmonary evaluation remained normal, rendering a diagnosis of systemic sarcoidosis unlikely. It was at this point in her disease course (about 2 years after onset) that the patient presented to our dermatology clinic, with clinical exam demonstrating 15% body surface area involvement (Fig 1). Considering earlier biopsy findings of mucin-positive granulomatous inflammation alongside annular plaque morphology, a diagnosis of GGA was made. The patient was hesitant to pursue additional systemic immunosuppressive therapy, and was unable to commit to frequent phototherapy sessions. Therefore, the patient was started on topical ruxolitinib 1.5% cream, to be applied twice daily to affected areas. After 12 weeks of consistent use, the patient achieved complete resolution of her lesions (Fig 2) with no adverse events. The patient remained mostly clear beyond 12 weeks, requiring ruxolitinib cream two–three times weekly to manage minor flares.

## Discussion

GGA is a rare disease with limited treatment options, especially for refractory disease. In this case, we highlight the efficacy of the topical Janus kinase (JAK) inhibitor, ruxolitinib 1.5% cream, in a patient who did not respond to treatment with topical corticosteroids or oral hydroxychloroquine. Recent research has elucidated the involvement of the JAK/signal transducers and activators of transcription (STAT) pathway in the pathogenesis of granuloma annulare, demonstrating constitutive activation of STAT1 and STAT3,^3^ in addition to upregulation of JAK2 and JAK3 in lesional skin.^4^ Consequently, systemic JAK inhibitors have emerged as promising treatment options for granuloma annulare, with several case reports describing the efficacy of oral tofacitinib,^3^^,^^5^ baricitinib,^6^ and upadacitinib.^7^ However, systemic immunosuppressive therapy with JAK inhibitors is not without risk, particularly in older patients and those with comorbidities.^8^ While there are reports documenting the efficacy of topical tofacitinib in treating granuloma annulare,^9^ its limited commercial availability restricts its use in clinical practice. Topical ruxolitinib, a selective JAK1 and JAK2 inhibitor, is well-tolerated and widely accessible.^10^ Unlike oral JAK inhibitors, the systemic absorption of topical ruxolitinib is low, with no reports of serious adverse events, such as acute cardiovascular events or cancer, in clinical trials.^10^ As such, topical ruxolitinib may be an appropriate, safe treatment option in cases when systemic therapy fails or is otherwise contraindicated. To our knowledge, this is the first report demonstrating the efficacy of ruxolitinib cream in GGA. Large-scale clinical trials evaluating its efficacy for granuloma annulare may be warranted.

## Conflicts of interest

None declared.
